# No Difference in Arousal or Cognitive Demands Between Manual and Partially Automated Driving: A Multi-Method On-Road Study

**DOI:** 10.3389/fnins.2021.577418

**Published:** 2021-06-10

**Authors:** Monika Lohani, Joel M. Cooper, Gus G. Erickson, Trent G. Simmons, Amy S. McDonnell, Amanda E. Carriero, Kaedyn W. Crabtree, David L. Strayer

**Affiliations:** ^1^Department of Educational Psychology, University of Utah, Salt Lake City, UT, United States; ^2^Department of Psychology, University of Utah, Salt Lake City, UT, United States

**Keywords:** heart rate, heart rate variability, detection response task, partial driving automation, applied cognition

## Abstract

**Introduction:**

Partial driving automation is not always reliable and requires that drivers maintain readiness to take over control and manually operate the vehicle. Little is known about differences in drivers’ arousal and cognitive demands under partial automation and how it may make it difficult for drivers to transition from automated to manual modes. This research examined whether there are differences in drivers’ arousal and cognitive demands during manual versus partial automation driving.

**Method:**

We compared arousal (using heart rate) and cognitive demands (using the root mean square of successive differences in normal heartbeats; RMSSD, and Detection Response Task; DRT) while 39 younger (*M* = 28.82 years) and 32 late-middle-aged (*M* = 52.72 years) participants drove four partially automated vehicles (Cadillac, Nissan Rogue, Tesla, and Volvo) on interstate highways. If compared to manual driving, drivers’ arousal and cognitive demands were different under partial automation, then corresponding differences in heart rate, RMSSD, and DRT would be expected. Alternatively, if drivers’ arousal and cognitive demands were similar in manual and partially automated driving, no difference in the two driving modes would be expected.

**Results:**

Results suggest no significant differences in heart rate, RMSSD, or DRT reaction time performance between manual and partially automated modes of driving for either younger or late-middle-aged adults across the four test vehicles. A Bayes Factor analysis suggested that heart rate, RMSSD, and DRT data showed extreme evidence in favor of the null hypothesis.

**Conclusion:**

This novel study conducted on real roads with a representative sample provides important evidence of no difference in arousal and cognitive demands. Younger and late-middle-aged motorists who are new to partial automation are able to maintain arousal and cognitive demands comparable to manual driving while using the partially automated technology. Drivers who are more experienced with partially automated technology may respond differently than those with limited prior experience.

## Introduction

Some commercially available vehicles with partial vehicle automation can support safe driving (e.g., Tesla-Autopilot, Nissan-ProPilot, Volvo-Pilot Assist, and Cadillac-Supercruise). However, partial vehicle automation is not always reliable, requiring drivers to maintain readiness to take over vehicle control at all times (SAE, 2016). If drivers’ cognitive demands during partially automated driving are different from manual driving mode, it may raise concerns about drivers’ cognitive readiness to take over, should automation fail. In studying cognitive demands during manual and partially automated driving, it is important to consider driver’s arousal (Yerkes and Dodson, 1908; Hebb, 1955; Broadhurst, 1959; Wekselblatt and Niell, 2015). The concern with partially automated technology is that it may lead to suboptimal arousal levels and cognitive demands resulting in poor driving performance. This motivated the study’s research question: Are there differences in drivers’ arousal and cognitive demands during manual versus partially automated driving? A comparison of physiological arousal and cognitive demands during the two modes of driving would help understand potential differences (or lack thereof) in cognitive demands and subsequent driving performance. The aim of the current study was to investigate whether there are differences in drivers’ arousal and cognitive demands during manual versus partial automation driving on real highways.

Only limited research is currently available to understand potential differences in drivers’ cognitive demands under partial vehicle automation. Based on classic cognitive models of attention (Kahneman, 1973; Wickens, 2002), *driving-related cognitive demands* are defined as all cognitive and mental processing resources required to perform a driving task. Some studies have suggested that there may be significant cognitive demands (e.g., workload) during automated driving compared to manual driving (Solís-Marcos et al., 2018; Kim et al., 2020). However, other research has found cognitive demands during partially automated driving may actually be reduced (Biondi et al., 2018; Heikoop et al., 2019; Zhai and Lu, 2019). By contrast, other research suggests little or no difference (Sibi et al., 2017; Stapel et al., 2019; Calvi et al., 2020; Várhelyi et al., 2020). Note that Stapel et al. (2019) found no difference in automation for inexperienced drivers; however, lower workload was found with experienced drivers. A variety of factors can help explain these inconsistent findings, including small sample sizes limiting the likelihood of detecting a true effect; testing only a single vehicle raising questions of ecological validity; and use of self-reports administered after driving raising concerns of retrospective report biases. Furthermore, most of the research on this topic has been restricted to samples of younger adults that limits the applicability of findings to the general public. Thus, a clear understanding of whether and how partially automated technology influences drivers’ arousal and cognitive demands are still lacking.

*Arousal* is a heterogeneous construct that involves general autonomic activation (e.g., Robbins and Everitt, 1995; Satpute et al., 2019). Heart rate is a biomarker of arousal and they are positively related (Berntson et al., 2007; Mauss and Robinson, 2010; Satpute et al., 2019). Heart rate is commonly used to measure driving-related arousal changes (Lohani et al., 2019) and it was used to operationalize arousal in the current study. Moreover, in real-world driving (for a review see, Lohani et al., 2019), multiple overlapping constructs dynamically change simultaneously and interdependently (e.g., workload, stress, boredom, distraction) resulting in net cognitive demands experienced by a motorist that can be broadly categorized along a low-to-high spectrum. Low cognitive demands may be represented by a combination of constructs such as drowsiness and boredom, while high cognitive demands may be represented by mental workload and stress (Lohani et al., 2019). Cognitive demands are associated with *cardiac vagal control*, which represents the influence of the vagus nerve on heart functioning (for a review, see Berntson et al., 2007; Laborde et al., 2017). Cardiac vagal control can be indexed by vagally-mediated *heart rate variability* (HRV), i.e., the temporal variability in adjacent heartbeats (Malik, 1996; Berntson et al., 2007). According to the neurovisceral integration model, the neural circuitry for cognitive and autonomic regulation has an overlapping neurovisceral mechanism (see Thayer et al., 2009; Smith et al., 2017), which can explain the coupling between cognitive demands and vagally-mediated HRV. Thus, vagally-mediated HRV can measure changes in cognitive demands, such as mental effort, workload, and attention (e.g., Mulder and Mulder, 1981; Mulder, 1985; Thayer et al., 2009).

Indeed, vagally-mediated HRV has been shown to provide a near-real-time objective measure of dynamic changes in cognitive demands, making it a suitable measure for applied driving research without disrupting the driving task (e.g., Lee et al., 2007; Liang et al., 2009; Mehler et al., 2011; Noda et al., 2015; Sugie et al., 2016; Heine et al., 2017; Piotrowski and Szypulska, 2017; Cisler et al., 2019; Lohani et al., 2019). Some of these studies found that cognitive state detection while driving was better when vagally-mediated HRV was utilized in addition to behavioral measures (e.g., Lenneman and Backs, 2009). Even though a variety of measures of vagally-mediated HRV have been used in applied research, the root mean square of successive differences in normal heartbeats (RMSSD) has been found to change more systematically with driving-related cognitive demands (Mehler et al., 2011; Heine et al., 2017), and thus was a suitable measure for the current study. In particular, RMSSD decreases with an increase in cognitive demands while driving (Mehler et al., 2011) due to the links between vagally-mediated HRV and neural activity associated with cognitive regulation (e.g., Thayer et al., 2009). Furthermore, a standard behavioral method to assess cognitive demands in driving research is the Detection Response Task (DRT; International Organization for Standardization [ISO], 2016). Performance on the DRT (International Organization for Standardization [ISO], 2016) is a measure of driving-related cognitive demands due to visual demands and driving difficulty (Bengler et al., 2012; Bruyas and Dumont, 2013; Cooper et al., 2016). An increase in driving-related cognitive demands is associated with increased reaction time performance and decreased hit-rate on the DRT (Young et al., 2013). At the same time, drowsiness and fatigue also increase reaction time on this task (Gershon et al., 2009). DRT was included as a behavioral measure of cognitive demands in the current study.

In prior research, we conducted a pilot study with 28 young drivers (*M_*age*_* = 29.29 years) new to partial automation who drove three partially automated vehicles on a flat and straight section of interstate highway (Lohani et al., 2020). In this pilot study, there were no differences between manual and partially automated driving modes across outcomes: heart rate, RMSSD, electroencephalogram (EEG) alpha, and theta power, and DRT performance. The current study is a new follow-up study designed to replicate and extend the earlier findings with a larger and more representative sample of younger and late-middle-aged drivers, partially automated vehicles (Cadillac, Nissan Rogue, Tesla, and Volvo), and sections of roadway. The current research measured heart rate to compare arousal and RMSSD and DRT performance to compare cognitive demands under manual versus partially automated driving.

Based on previous work, the current study considered three alternative hypotheses. First, if partially automated driving leads to high arousal and high cognitive demands (e.g., workload and stress; Solís-Marcos et al., 2018; Kim et al., 2020), then, a significant increase in heart rate, a decrease in RMSSD, and an increase in the DRT reaction time rate would be expected when compared to manual driving. Second, if partially automated driving leads to low arousal and low cognitive demands (e.g., drowsiness and boredom; Biondi et al., 2018; Heikoop et al., 2019; Zhai and Lu, 2019), then a significant decrease in heart rate, an increase in RMSSD, and an increase in DRT reaction time would be expected when compared to manual driving. Finally, if arousal and cognitive demands are similar under manual and partially automated driving, then no differences in the outcome measures would be expected (e.g., Sibi et al., 2017; Stapel et al., 2019; Calvi et al., 2020; Lohani et al., 2020; Várhelyi et al., 2020). This prediction of the null hypothesis has a compelling rationale and a meaningful interpretation that would imply that manual and partially automated modes are comparable in arousal and cognitive demands. However, some limitations (e.g., small sample size, a non-representative sample of people and vehicles, and inadequate statistics) can hamper the ability to interpret evidence for a null hypothesis adequately. Therefore, we designed the current study to allow for a fair test of the null hypothesis by testing a larger and more representative sample of drivers and vehicles. We bolstered the interpretation by conducting the classic null-hypothesis significance testing and a Bayesian alternative (Kruschke, 2011) to meaningfully interpret whether the current dataset supported the null or the alternative hypothesis.

## Materials and Methods

### Participants

A total of 71 adults with no prior experience with partially automated vehicles participated in this study. 39 participants were younger (21–42 age range, *M_*age*_* = 28.82 years, *SD*_*age*_ = 6.41, 13 females). 32 participants were late-middle-aged (43–64 age range, *M_*age*_* = 52.72 years, *SD*_*age*_ = 6.33, 12 females). The study protocol was in accordance with the Institutional Review Board at the University of Utah. Participants had no previous experience with partial automation, had a valid driver’s license, no at-fault accidents in the past 2 years, drove at least an average of 10 h per month, had no history of neurological disorders or heart conditions, and were not pregnant. In addition, participants were required to pass a 20 min online defensive driving course and certification test. Upon arrival in the lab, eligible participants were allowed to participate in the study only if they had slept at least 6 h the previous night and had their blood alcohol level at 0.0%, which was verified using a BACtrac breathalyzer.

### Measures

Psychophysiological data was continuously sampled at 2,000 Hz using a portable wireless physiology system (Smart Center, Biopac System Inc., United States) and Acqknowledge software (Biopac System Inc., United States). This setup allowed real-time data quality monitoring while participants drove on the highway.

#### Electrocardiography

The electrical activity of the heart was recorded by using an electrocardiogram (ECG). After cleaning the site, standard disposable electrodes were placed on the right collar bone and the left and right end of the ribcage (Lead II configuration; Berntson et al., 2007). During data collection, the ECG was monitored by a research assistant who sat in the front passenger seat. Any noticeable movements that could add artifacts (e.g., sneezes and itch) were marked.

Standardized methods in accordance with recommended guidelines for ECG data were followed (e.g., Malik, 1996; Berntson et al., 1997, 2007; Peltola, 2012; Shaffer et al., 2014; Laborde et al., 2017). Post data collection, the ECG data was processed using AcqKnowledge software (Biopac System Inc., United States). The raw data was bandpass filtered at 1 and 35 Hz cutoffs. The software was used to detect R-wave peaks. All R-wave peaks were visually inspected for accurate detection and manually corrected if the software marked improbable values. This included any artifacts generated by facial or head movements (e.g., yawns or checking blind spots while driving). After data cleaning, data were processed to calculate RMSSD and heart rate for manual and partially automated driving tasks for each vehicle operated by each participant. Based on the recommended guidelines (Malik, 1996; Berntson et al., 1997; Laborde et al., 2017), RMSSD was calculated using the same length epochs (1 min) for the pre-condition baseline and the main-condition periods (see the procedure for details). The epochs were then averaged over the entire period to get average RMSSD values during the pre-condition and main-condition periods. For RMSSD and heart rate data, any values that exceeded three standard deviations from the mean of normal distribution were removed before analyses.

#### Detection Response Task (DRT)

To probe drivers’ cognitive demand, participants were asked to perform a vibrotactile detection task, DRT. In line with the ISO 17488 guidelines (2016), a vibrotactor (a small vibration motor) emitted a small vibration stimulus, similar to a vibrating cell phone. This stimulus was presented pseudo-randomly every 3–5 s. Participants wore a vibrotractor that was taped to their left biceps. A microswitch was attached to either the index or middle finger of the left hand, which participants could press against the steering wheel to respond to the onset of the stimulus. Instead of the standard left collarbone placement, the left bicep was used to avoid any potential interference with the ECG signal. A similar approach has been successfully used in past work (e.g., Lohani et al., 2020). Participants’ goal was to respond to the stimulus onset as quickly as possible while driving (with priority always given to safe driving practices). Response time in milliseconds was recorded for each stimulus. The vibration stimulus was set up to turn off after 1 s.

The average reaction time performance on DRT was calculated for each participant in each vehicle in manual and partially automated driving tasks. Any values that exceeded three standard deviations from the mean of normal distribution were removed before analyses. The hit-rate performance for the current sample was at a ceiling level (∼95% and above). The average proportion of hit-rate during manual driving was 96% (*SE* = 0.003) and during partially automated driving was 95% (*SE* = 0.004). Because of a lack of variance in performance, the hit-rate was not further analyzed.

#### Self-Reported Experiences With Automation

After driving partially automated vehicles, participants responded to a list of questions about their experiences and attitudes about partially automated vehicles. An example item was “I was anxious and nervous when the automated driving systems were on.” Participants indicated their agreement to the following statements using a 5-point scale anchored by completely disagree to completely agree.

### Vehicles

We examined a representative sample of commercially available vehicles for this study. A 2018 Cadillac CT6, 2019 Nissan Rogue, 2018 Tesla Model 3, and a 2018 Volvo XC90 were used in this study. Each of these vehicles was equipped with the partial vehicle automation that centered the vehicle within the lane (e.g., Lane Centering) and maintained the following distance and speed (e.g., Adaptive Cruise Control). These features, when activated together, meet the definition of partial automation (SAE, 2016).

### Procedure

Participants were sent a training document and a short video about the partial automation features of the vehicle they would drive for the day of the visit. Upon arrival in the lab, after completing the consent form and inclusion criteria testing, participants were set up for ECG data collection. Next, a research assistant directed the participant to the designated vehicle in the parking lot close to the lab. Participants were instructed that they were driving commercially available vehicles, and we were interested in examining the vehicular systems on real roads. They were instructed to operate the vehicle as they would usually drive on the road.

Before driving the vehicle, participants would familiarize themselves with the vehicle and get trained on steps to activate its partial automation features. They engaged the partially automated systems during the training phase, and only when they were comfortable operating the vehicle was the main part of the study started. Participants were asked to keep their hands on the wheel and monitor the road (as recommended by most vehicle manufacturers). In the partial automation condition, participants always had the automation engaged. In rare instances, participants took control of the vehicle to pull over for emergency vehicles, debris on the road, and construction. Participants drove on the same road in manual and partial automation (counterbalanced). As soon as possible, partial automation was re-engaged. It is important to note that participants drove on the same road in both manual and partial automation (counterbalanced), so driving conditions were equated as best as possible. Moreover, any section of the drive where the driver had to deal with an event (e.g., pulling over for an emergency vehicle) were excluded from the analyses (in both manual and partial automation) to ensure a fair comparison.

Participants were also fitted with the DRT equipment and trained on how to perform the DRT task while driving. ECG data were monitored for quality. Next, they drove on a training route with the research assistant in the passenger seat to ensure that the participant could engage and disengage partial automation, change lanes, and control the vehicle’s speed. After confirming that participants understood how to use the partial automation and the DRT task while driving safely, the study’s testing phase was initiated.

For the testing phase, all participants drove comparable distances on two highways (I-15 and I-80) in two modes (manual and partial automation), with the order partially counterbalanced across participants (leading to 4 experimental sessions in each vehicle). The Average Annual Daily Travel (AADT) for I-15 (N/S bound 4–5 lanes in each direction separated by a median) is 175,000 vehicles and for I-80 (E-W bound 2–3 lanes in each direction separated by a median) is 19,000 vehicles (Utah Department of Transportation). Because there were no significant differences in outcomes between the two highways, the data were averaged for the two highways. The manual and partially automated conditions were also counterbalanced to control for any unsystematic differences.

Each of the four experimental sessions was about 20 min and began once the vehicle was at the posted speed limit. The first 2 min of each session were used as the *pre-condition baseline* measurement. The following 18 min of the experimental session were the *main condition* during which the DRT task was performed while participants drove in manual or partial automation conditions (depending on the order). Participants received a short rest break between each of the four experimental sessions. The average value of RMSSD (calculated by averaging 1 min epochs) in the pre-condition baseline period was subtracted from the average value of the main condition to account for any baseline differences within participants. The average heart rate in the pre-condition baseline was subtracted from the main condition. After accounting for baseline differences and collapsing across the two highways, there were two average values for each vehicle driven in manual and partial automation.

### Data Analysis Plan

The primary research focus was to examine how automation (manual versus partial automation) affects driver arousal and cognitive demand. To address this question, two analytical approaches were adopted – linear mixed-effects models and Bayes Factor analysis. For each of the outcome measures, a linear mixed-effects model was run with three fixed factors, automation (manual and partially automated), age (younger or late-middle-aged), and vehicle (Cadillac, Nissan Rogue, Tesla, and Volvo) and participants as the random intercept. Note that preliminary analyses were performed to determine if time should be included as a factor in the model. We found that including time as a factor in the model did not explain any significant variability. In contrast to automation condition and age, we had no theoretical or empirical rationale to include time in the models. Thus, to keep a parsimonious model, time was not included in the model because it did not explain significant variability in the outcome variables.

Next, Bayes Factor analysis was conducted to adopt a Bayesian alternative to evaluate how meaningful a significant difference or a lack of significant difference was between the manual and partial automation for each of the outcome variables. This was done by comparing a full model with automation, age, vehicle as predictors to a restricted model without automation (e.g., Kruschke, 2011). The Bayes Factor value is the ratio of the marginal likelihoods of the full model and the restricted model (Lee and Wagenmakers, 2014). The resulting Bayes factor value was interpreted using the classification scheme such that a value higher than 1 is interpreted as evidence in favor of the alternative hypothesis. A Bayes Factor value between 1 and 3 provides anecdotal evidence, 3–10 provides moderate evidence, 10–30 provides strong evidence, 30–100 provides very strong evidence, and a value more than 100 is extreme evidence in favor of the alternative hypothesis. By contrast, a value lower than 1 is interpreted as evidence in favor of the null hypothesis. Correspondingly, a value of 1–0.33 provides anecdotal evidence, 0.33–0.1 provides moderate evidence, 0.1–0.03 provides strong evidence, 0.03–0.01 provides very strong evidence, and a value less than 0.01 provides extreme evidence in favor of the null hypothesis (Lee and Wagenmakers, 2014; Quintana and Williams, 2018). All analyses were done by using the R language for statistical computing (R Core Team, 2020). Mixed models were fit using the *lme4* package (Bates et al., 2015), and Bayes Factor values were calculated via the *BayesFactor* package (Morey et al., 2018).

## Results

**FIGURE 1:**
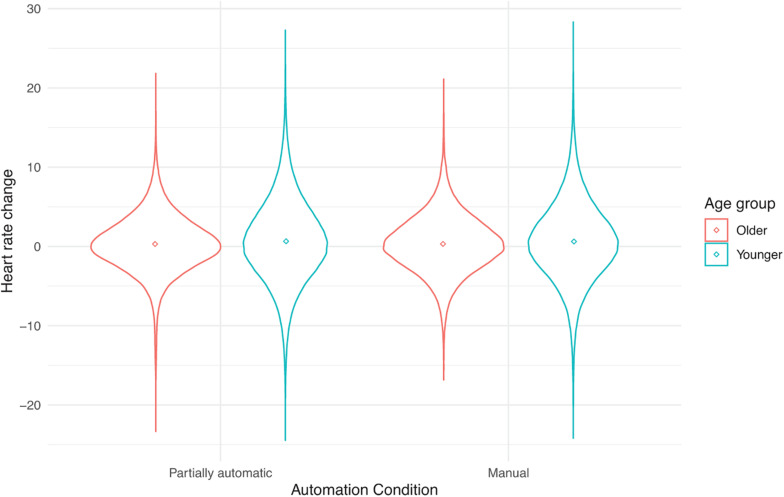
Heart rate change from baseline as a function of automation (manual and partial automation) and age (younger or older).

**FIGURE 2:**
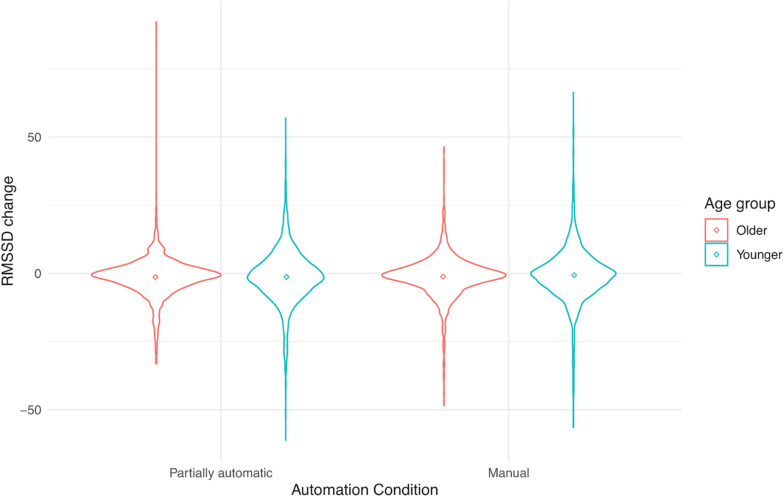
RMSSD change from baseline as a function of automation (manual and partial automation) and age (younger or older).

**FIGURE 3:**
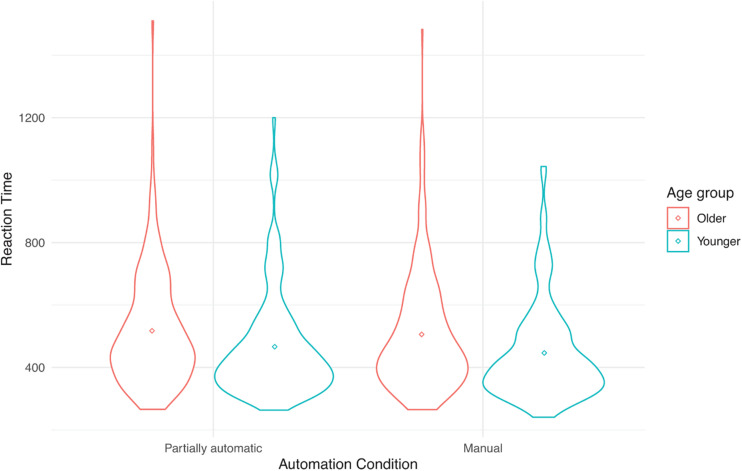
Reaction time (in ms) performance on the Detection Response Task as a function of automation (manual and partial automation) and age (younger or older).

See Table 1 for descriptive statistics for the three outcome variables as a function of age and vehicle. Figures 1–3 present individual data points to complement the descriptive tables. These include violin plots that are similar to box plots, but in addition, they have the kernel probability density of all the observed data. The mean values are also indicated by a dot in the center of each distribution.

**TABLE 1:** Means (and Standard Error) for heart rate, RMSSD, and DRT reaction time as a function of automation (manual and partial automation), age (younger or older), and vehicle (Cadillac, Nissan Rogue, Tesla, and Volvo).

Measure	Automation	Young				Old			
		
		Cadillac	Nissan	Tesla	Volvo	Cadillac	Nissan	Tesla	Volvo
**Heart rate**	Manual	0.49 (0.32)	0.37 (0.32)	0.76 (0.34)	0.81 (0.33)	0.58 (0.34)	−0.11 (0.34)	0.38 (0.33)	0.60 (0.34)
	Partial automation	0.26 (0.33)	1.05 (0.32)	0.38 (0.33)	0.48 (0.33)	0.30 (0.34)	0.27 (0.33)	0.33 (0.34)	0.55 (0.34)
**RMSSD**	Manual	−0.45 (0.96)	−0.28 (0.96)	0.07 (1.01)	−2.02 (0.98)	0.68 (1.02)	−0.64 (0.98)	−2.72 (0.96)	−0.96 (1.00)
	Partial automation	−0.49 (0.97)	−1.90 (0.96)	−0.66 (0.99)	−2.36 (0.97)	−1.83 (1.02)	−0.25 (0.98)	−1.81 (1.0)	−1.71 (1.0)
**DRT reaction time**	Manual	437 (28.5)	443 (28.9)	455 (29.2)	480 (28.9)	498 (31.1)	495 (30.4)	498 (30.5)	523 (31.1)
	Partial automation	459 (28.5)	463 (28.9)	468 (29.2)	489 (28.9)	506 (31.4)	529 (30.4)	508 (30.5)	518 (31.1)

Age differences and gender differences were examined during the pre-condition baseline and main condition. In the pre-condition baseline values of RMSSD, there were significant gender and age-related differences with higher mean values for males (*M* = 29.5, *SE* = 1.76) than females (*M* = 22.7, *SE* = 2.27), *t*(69) = 2.38, *p* = 0.02, and higher RMSSD values for younger (*M* = 30.2, *SE* = 1.96) than late-middle-aged (*M* = 21.9, *SE* = 2.10) drivers, *t*(69) = 2.89, *p* = 0.01. However, after accounting for pre-condition values (average main condition – average pre-condition baseline values), neither gender differences (*p* = 0.19) nor age differences (*p* = 0.89) were significant. We did not have specific hypotheses for gender-related differences in driving manual and partially automated vehicles. Thus, gender was not included as a factor in the models to test the study’s research questions.

### The Effect of Automation on Heart Rate

A linear mixed-effects model was run with a baseline-corrected heart rate as the outcome. Neither the main effect of automation, *F*(1, 320.48) = 0.05, *p* = 0.82, age, *F*(1, 60.71) = 0.69, *p* = 0.41, nor vehicle, *F*(3, 355.24) = 0.42, *p* = 0.74 were significant. Likewise, none of the 2-way or 3-way interactions were significant.

### The Effect of Automation on RMSSD

A linear mixed-effects model examined how RMSSD varied as a function of automation (manual and partially automated), age (young or late-middle-aged), and vehicle (Cadillac, Nissan Rogue, Tesla, and Volvo), and participants as the random intercept. No main effect of automation was found, *F*(1, 321.99) = 1.76, *p* = 0.19. In addition, no main effect of age, *F*(1, 60.96) = 0.04, *p* = 0.84, or vehicle was found, *F*(3, 358.84) = 1.45, *p* = 0.23. Neither the automation by age by vehicle interaction nor any of the 2-way or 3-way interactions were significant.

### The Effect of Automation on DRT Reaction Time

A linear mixed-effects model with reaction time as the outcome did not have a significant main effect of automation, *F*(1, 325.77) = 2.76, *p* = 0.10. Similarly, neither the main effects of age, *F*(1, 73.77) = 1.77, *p* = 0.19, nor vehicle, *F*(3, 337.02) = 1.67, *p* = 0.17 were significant. The 2-way and 3-way interactions were also not significant.

### Bayes Factor Analyses

**FIGURE 4:**
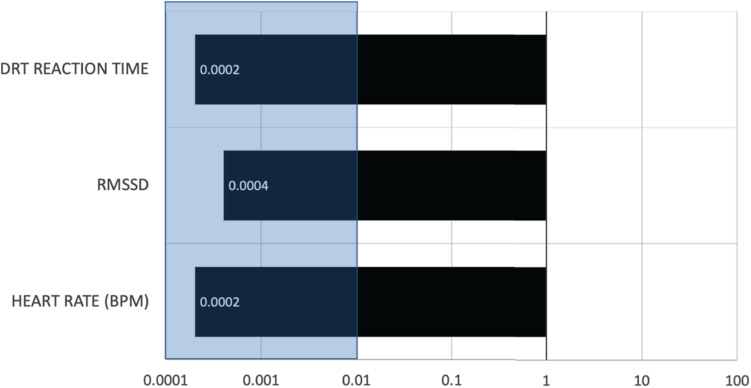
The Bayesian values were plotted for the three outcome variables. A value less than 0.01 (shaded region) provides extreme evidence in favor of the null hypothesis (Lee and Wagenmakers, 2014).

In order to examine the effect of automation on RMSSD, heart rate, and DRT reaction time, for each of these outcomes, a Bayes factor analysis was conducted by running a full model with main effects and interactions of automation, age, vehicle, and participants as the random intercept. Next, a restricted model was run without automation with main and interaction effects of age and vehicles as predictors and participants as the random intercept. These full and restricted models were compared to calculate a Bayes Factor of 0.0002, 0.0004, and 0.0002 for heart rate, RMSSD, and DRT reaction time, respectively (see Figure 4). According to the Lee and Wagenmakers (2014) classification scheme for interpreting Bayes factors, these values suggest extreme evidence that favors the null hypothesis for the effect of automation on heart rate, RMSSD, and DRT reaction time.

### Self-Reported Driving Experience

All participants drove in the partially automated conditions and their experiences on a 5-point scale (completed disagree to completely agree) were analyzed by comparing the responses to the midpoint. The results are reported in Table 2. Participants could relax, but relative to manual driving, they were neither less stressed nor bored when the automated driving systems were activated. Participants reported not engaging in unrelated activities while driving the automated systems, such as daydreaming. Participants were well-calibrated in their trust in the automated features of the vehicles. On the one hand, they believed that it made traveling safer and enjoyable, and they were not more anxious or nervous while driving it relative to manual driving. At the same time, they showed restraint when the driving conditions were challenging (e.g., curvy/hilly roads and heavy traffic).

**TABLE 2:** Results from the driving experiences questionnaire.

Items	Finding	Statistic	Mean (SE)
**The driving experiences related questions**			
I was able to relax when the automated driving systems were on	Agree	*t*(210) = 5.32, *p* < 0.001	3.44 (0.08)
I was able to engage in more activities unrelated to driving when the automated driving systems were on	Disagree	*t*(210) = 6.35, *p* < 0.001	2.43 (0.09)
The automated driving system made traveling boring for me	Disagree	*t*(210) = 8.95, *p* < 0.001	2.26 (0.08)
The automated driving systems made traveling safer	Agree	*t*(210) = 5.46, *p* < 0.001	3.39 (0.07)
I was anxious and nervous when the automated driving systems were on	Disagree	*t*(210) = 5.69, *p* < 0.001	2.51 (0.09)
The automated driving system made traveling more enjoyable	Agree	*t*(210) = 6.14, *p* < 0.001	3.47 (0.08)
The automated driving system took the fun out of driving	Disagree	*t*(210) = 5.59, *p* < 0.001	2.49 (0.09)
The automated driving system allowed me to think and daydream	Disagree	*t*(210) = 4.53, *p* < 0.001	2.61 (0.09)
I was uncomfortable relinquishing control of the vehicle to the automated driving system on curvy and hilly roads	Agree	*t*(210) = 5.31, *p* < 0.001	3.48 (0.09)
I was uncomfortable relinquishing control of the vehicle to the automated driving systems in heavier traffic	Agree	*t*(210) = 3.08, *p* = 0.002	3.28 (0.09)
I was concerned that the automated driving systems would shut off unexpectedly	Not sig.	*t*(210) = 0.10, *p* = 0.919	2.99 (0.09)
The automated driving system reduced the stress of driving	Not sig.	*t*(210) = 1.84, *p* = 0.067	3.16 (0.07)
**Intentions to use and purchase automated driving systems**			
I would not feel comfortable using automated driving systems on most roads	Disagree	*t*(210) = 7.31, *p* < 0.001	2.39 (0.08)
If I was tired or distracted, I would rely heavily on automated driving systems	Disagree	*t*(210) = 2.46, *p* = 0.015	2.78 (0.09)
I would utilize the automated driving systems in a vehicle as much as possible	Agree	*t*(210) = 8.41, *p* < 0.001	3.68 (0.08)
I would not feel comfortable using the automated driving systems in a vehicle without monitoring it closely	Agree	*t*(210) = 13.16, *p* < 0.001	4.00 (0.08)
If I can afford it, I am going to buy or lease a car with automated driving systems	Agree	*t*(210) = 7.84, *p* < 0.001	3.67 (0.09)
I am going to make sure that the next car I buy, or lease has automated driving systems	Not sig.	*t*(210) = 1.84, *p* = 0.067	3.15 (0.08)

## Discussion

### Lack of Differences Between Manual and Partially Automated Modes

In order to examine the possible impact of partially automated technology on drivers’ arousal and cognitive demand, it is necessary to have sensitive near real-time measures that can detect changes in real-world driving tasks. The current study used heart rate (an arousal measure), RMSSD (a heart rate variability based cognitive demands measure), and DRT (a behavioral performance task based cognitive demands measure) to compare differences in drivers’ arousal and cognitive demands during manual versus partially automated driving. To our knowledge, this study is the first effort to examine the partial vehicle automation on arousal and cognitive demands with a representative sample of younger and late-middle-aged drivers and vehicles on real highways while their psychophysiological and behavioral responses were assessed in real-time.

The results suggested that there were no differences in heart rate and its variability or DRT reaction time performance between manual and partially automated modes of driving either in younger or late-middle-aged adults across the four test vehicles. A Bayes Factor analysis on heart rate, RMSSD, and DRT reaction time data showed *extreme evidence in favor of the null hypothesis*, suggesting that drivers new to partial automation maintain comparable levels of arousal and cognitive demands during manual and partially automated driving as no evidence of under or over-arousal and cognitive demands was found. In another forthcoming paper (McDonnell et al., in review), EEG alpha power and frontal theta (a central physiology-based index of visual engagement and mental workload) were also found to show no significant difference between manual and partially automated modes. Past research on potential cognitive differences between manual and partial automation has been mixed. Various methodological limitations can explain these mixed findings, such as limited sample size, use of only self-report measures, and the use of driving simulators or a single-vehicle. The current study allowed for a fair test of the null hypothesis by testing a large representative sample of drivers and vehicles with sufficient sample size and a combination of reliable physiological and behavioral measures.

The current findings support and extend previous research that has found no difference between manual and partial automation (e.g., Sibi et al., 2017; Stapel et al., 2019; Calvi et al., 2020; Lohani et al., 2020; Várhelyi et al., 2020). Moreover, the pilot study that examined young drivers operating three vehicles on the highway also showed a similar pattern (Lohani et al., 2020). Similar to the current study, the pilot study found no differences across outcomes (heart rate, RMSSD, EEG alpha and theta power, and DRT performance). Bayes Factor analysis had suggested that there was strong evidence that arousal and cognitive demands did not differ during manual and partially automated driving (Lohani et al., 2020). With a single measure, null results are harder to interpret, but replicable effects with multiple reliable outcomes provide a more convincing interpretation of the null hypothesis. Taken together, these findings provide strong evidence that arousal and cognitive demands are similar during manual versus partially automated conditions for drivers who are new to partially automated technology.

A lack of differences between manual and partially automated modes also suggests that the cognitive demands imposed during manual driving are comparable to those imposed by monitoring partial automation. Based on the Neurovisceral Integration model (Thayer et al., 2009), these findings would imply that neural activity associated with cognitive functioning during manual and partially automated driving is comparable. Self-reports from participants suggested that participants were cognitively engaged in the driving process. This may be because participants still have to monitor ongoing traffic conditions and maintain cognitive readiness to take control of the vehicle. Interestingly, the age group did not lead to any differences in heart rate and RMSSD (after accounting for pre-condition baseline) or DRT reaction time. This implies that late-middle-aged drivers are able to use partially automated technology similar to their younger counterparts and that they can benefit from assistance provided by partially automated technology without additional cognitive costs. While this study investigated drivers between 21 and 64 years of age, future research should examine potential differences in teenage drivers and drivers older than 64 years of age to better understand driving partial automation vehicles across the lifespan.

### Limitations and Outstanding Questions

There are a few limitations of the current study. First, this study focused on drivers with no prior experience with partial driving automation. Drivers that are more experienced with partially automated technology may respond differently to automated vehicles than those with limited prior experience. The perceived workload was reduced for automation-experienced drivers, while it did not change from the manual mode for inexperienced drivers (Stapel et al., 2019). Motorists may learn to accept and trust the automation technology with additional driving experience (Beggiato et al., 2015). Experienced drivers may get better at calibrating their trust after understanding the automation system’s limitations (Walker et al., 2018). However, it is also possible that more trusting participants increase their reliance on automation (Walker et al., 2019), resulting in poor readiness to switch from automated to manual driving safely. More work is needed to explore any associated risks.

Second, we had a research assistant sit in the passenger seat next to the participant for safety reasons, and we cannot rule out that this presence may have impacted drivers’ performance and physiology. However, any confounding effects of social presence were constant in both manual and partial automation conditions across all the participants. Third, this study was not explicitly designed to test gender differences. Factors such as the different phases of the menstrual cycle were not recorded or accounted for and could affect the RMSSD data. Future work is needed to understand the effect this may have on RMSSD. Fourth, this study did not examine traffic conditions, and low and high traffic demands could moderate the outcomes. Finally, it is possible that constantly transitioning between manual and partially automated driving modes could be a demanding task for some motorists. For instance, one study found that older adults were slower at switching between manual and partially automated modes (Wu et al., 2019). Future research should examine transition-related driving demands on motorists while driving partially automated vehicles.

### Benefits and Challenges of Adopting HRV Indices in Driving Safety

With advances in methodological developments, it is now possible to collect high-quality psychophysiological data outside traditional lab settings from research-grade equipment at a low cost. This study conducted on real roads highlights the applicability of heart rate variability to real-world automation driving research. However, some caution is warranted while interpreting HRV measurement. As is true for most psychophysiological measures, HRV does not have a one-to-one correspondence with a single psychological construct (e.g., Cacioppo and Tassinary, 1990). HRV may be sensitive to many cognitive factors that may occur in real-world driving contexts. For example, over time, drivers may become more relaxed or disengaged with the driving task and both scenarios would lead to a lower-arousal and a corresponding increase in HRV indices (Jasper et al., 2016). Furthermore, there may be other factors (e.g., Laborde et al., 2017) while driving that can influence HRV measurements, such as driving task-related factors (e.g., bad traffic), driver-related factors (e.g., a driver has irregular heartbeats or is on psychotropic medications), or concurrent activities (talking, smoking, or caffeine intake). Because such factors may influence HRV indices and may vary across driving conditions; thoughtful analyses of such effects in conjunction with other measures (such as behavioral performance) is crucial for accurate interpretations.

A challenge with HRV measurement is that it is susceptible to artifacts that can result in inaccurate values. Some analytic approaches have adopted shorter segments (e.g., 30 s) of data to detect momentary effects of driving-related workload (Stuiver et al., 2014). While preliminary solutions to handle noise and artifacts have been proposed (e.g., Nowara et al., 2018; van Gent et al., 2019; Brown et al., 2020), more work is needed to develop near-real-time artifact detection methods that are amenable to driving task-related changes. As in the current study, we argue that a multi-method approach provides a more accurate interpretation of cognitive demands in applied settings. Several efforts in driving research have proposals to assess cognitive states using a multi-method approach that seems promising (Fu et al., 2016; Brouwer et al., 2017; Aricò et al., 2018; Haouij et al., 2018; Paredes et al., 2018; Rastgoo et al., 2018). The current findings imply that RMSSD is a suitable measure that could be utilized in multi-method studies to evaluate drivers’ cognitive demand.

## Conclusion

This investigation was conducted on real roads with a large and representative sample of younger and late-middle-aged motorists to compare manual and partially automated driving. The current findings show important evidence of no difference in arousal and cognitive demands (extreme evidence in favor of the null hypothesis), suggesting that drivers maintain similar levels of arousal and cognitive demands during manual and partially automated driving. These theoretically relevant results imply that younger and late-middle-aged motorists who are new to partial automation may maintain arousal and cognitive levels comparable to manual driving. This research extends a growing literature highlighting the applicability of heart rate variability to real-world automation research.

## Data Availability Statement

The raw data supporting the conclusions of this article will be made available by the authors, without undue reservation.

## Ethics Statement

The studies involving human participants were reviewed and approved by the Institutional Review Board, University of Utah. The patients/participants provided their written informed consent to participate in this study.

## Author Contributions

DS and JC conceived the study idea and design. ML, GE, TS, AC, and KC were involved in data collection and processing. ML analyzed the data and wrote the first draft of the manuscript. DS, JC, and AM provided suggestions to improve the manuscript. All authors contributed to the article and approved the submitted version.

## Conflict of Interest

The authors declare that the research was conducted in the absence of any commercial or financial relationships that could be construed as a potential conflict of interest.
